# Complete mitochondrial genome data and phylogenetic analysis of *Papilio macilentus* Janson, 1877 (Lepidoptera: Papilionoidea: Papilionidae)

**DOI:** 10.1080/23802359.2024.2351536

**Published:** 2024-05-13

**Authors:** Yun-Fei Wu, Wei-Hao Yang, Ai Jin, Yan Dong, Jia-Jia Wang, Li-Xin Zhu

**Affiliations:** College of Biology and Food Engineering, Chuzhou University, Chuzhou, China

**Keywords:** *Papilio macilentus*, next-generation sequencing, mitogenome, phylogeny

## Abstract

In the present study, the complete mitochondrial genome (mitogenome) of the *Papilio macilentus* (Lepidoptera: Papilionoidea: Papilionidae) was sequenced by next-generation sequencing method. The mitochondrial genome is a circular DNA molecule of 15,264 bp in size with 80.7% AT content, including 37 genes (13 protein-coding genes, 2 rRNA genes, and 22 tRNA genes), and a long non-coding region (Control region). All protein-coding genes are initiated by ATN codons, and terminated with TAA, TAG, or single T. All tRNAs can be folded into common clover leaf secondary structure, except *trn-S1*. Phylogenetic analyses based on 13 protein-coding genes and 2 rRNA genes using maximum likelihood and Bayesian inference confirmed that *P. macilentus* and *Papilio memnon* are clustered into a clade, and revealed the relationships between Papilionini, Troidini, Teinopaippini and Leptocircini.

## Introduction

**Figure 1.**
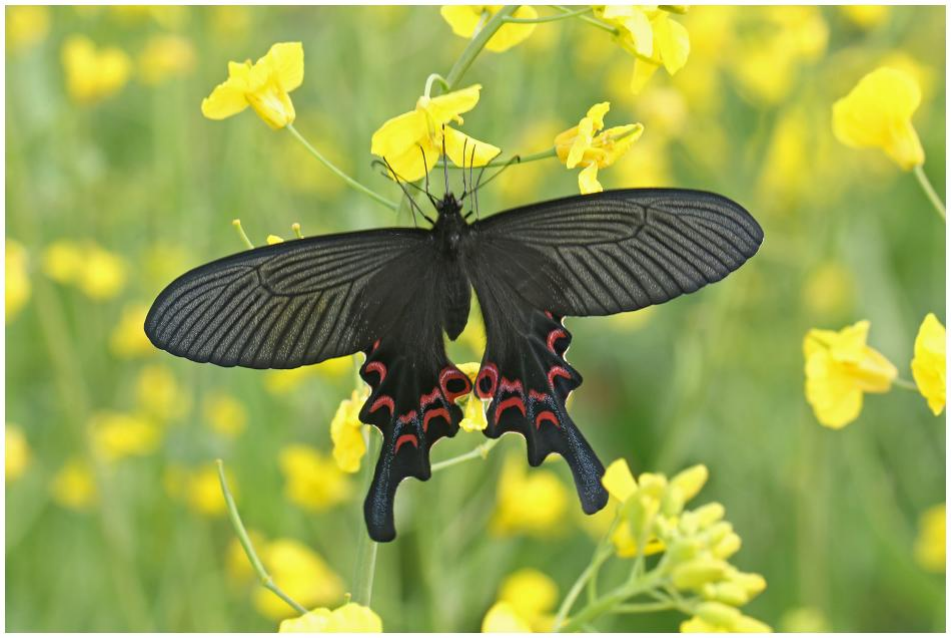
The adult specimen of *Papilio macilentus*. Photographed and processed by Yun-Fei Wu.

Papilionidae has attracted more attention due to extensive morphological diversity and their colorful wing patterns, and have been widely studied regarding ecological adaption, phylogeny, genetics, and evolution (He et al. 2022). *Papilio macilentus* Janson, 1877 belongs to Papilionidae, and is mainly distributed in China, Korea, Japan, and Eastern Siberia, more than one or two generations a year in China, larvae mainly feed on *Zanthoxylum* and *Ruta*, and adults are visible from April to August in Anhui province, China (Wu 2001). As a member of the ornamental butterfly species, *P. macilentus* is morphologically similar to *Papilio bianor*, but *P. macilentus* shows more narrow hind wings, and the outer margin area is different in red spots (Figure 1). Relatively little attention has been paid to *P. macilentus*, possibly because the population of the *P. macilentus* is significantly smaller in nature. In order to fill in the gap and clarify the mitochondrial genome (mitogenome) characteristics of *P. macilentus*, we have sequenced and annotated the complete mitogenome of *P. macilentus* by next-generation sequencing method, which would be useful for species identification, phylogenetic analysis and conservation of this species.

## Materials and methods

In this study, total genome DNA was extracted from the male adult leg of *P. macilentus* which was collected from Chuzhou City (118.295 E, 32.271 N), Anhui Province, China in April 2017. The total DNA was extracted using a DNeasy© Tissue Kit (Qiagen, Hilden, Germany). The genome was sequenced by the next-generation sequencing method with the Illumina Hiseq 2500 platform. The mitogenome was assembled using Geneious Primer v.2021.2.2 (Kearse et al. 2012) and annotated with MITOS server (Bernt et al. 2013) and NCBI BLAST (https://blast.ncbi.nlm.nih.gov). The specimen and genome DNA were deposited at −80 °C in the Animal Collection of Chuzhou University with the voucher number CHZU-LPP-0254 (Yan Dong, dongyan_bio@126.com). To investigate the phylogenetic status of *P. macilentus* in Papiliononae, 46 currently available complete mitogenomes of Papiliononae retrieved from GenBank were used in phylogenetic analysis, two Parnassiinae insects (*Sericinus montela*, HQ259122 and *Zerynthia polyxena*, MK507888) were used as outgroup. The phylogenetic tree was reconstructed with maximum likelihood (ML) and Bayesian inference (BI) methods based on the 13 protein-coding genes (PCGs) and 2 rRNAs by IQ-TREE v2.1.3 (Minh et al. 2020) and PhyloBayes MPI 1.8c (Lartillot et al. 2013), respectively. The number of bootstrap replicates was set to 1000 with automatic model prediction in ML analyses. The site-heterogeneous mixture model (CAT + GTR) was implemented in BI analyses. The consensus tree was computed with a burn-in of 25% of sampled values. Each gene was aligned using MAFFT 7 (Katoh K & Standley, 2013) and trimmed with trimAl 1.4.1 (Capella-Gutiérrez et al. 2009), then concatenated individual genes using MEGA X (Kumar et al. 2018).

## Results

**Figure 2.**
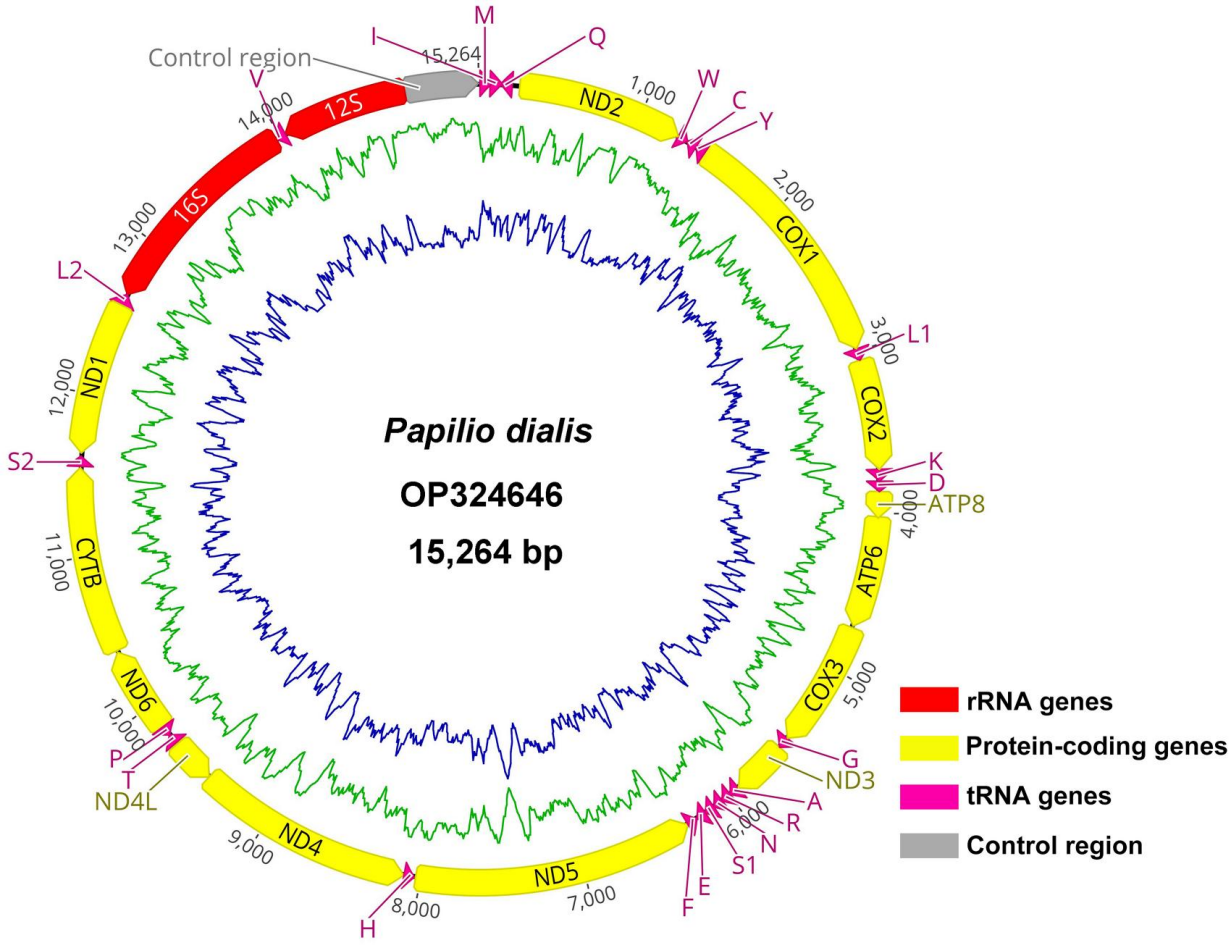
Circular map of the *Papilio macilentus* mitochondrial genome.

In this research, about 6.4 Gb of clean data were obtained and produced a final mitogenome for *P. macilentus* with an average sequencing depth of 1,374× (Figure S1). After checking and annotating, the complete mitogenome of *P. macilentus* is 15,264 bp (Figure 2), which is comparable to the sizes of previously documented mitogenomes of Papiliononae species, ranging from 14,642 bp of *Graphium antiphates* (MN013005) to 16,094 bp of *Papilio maraho* (FJ810212; Wu et al. 2010; Liu et al. 2020), the gene order of the *P. macilentus* mitogenome is identical to other commonly sequenced Papilionidae species. The mitogenome of *P. macilentus* nucleotide composition was A (39.7%), T (41.0%), G (7.4%), C (11.9%), and A + T content (80.7%). The complete mitogenome was composed of 37 typical mitochondrial genes (13 PCGs, 22 transfer RNA (tRNA) genes, two ribosomal RNA (rRNA) genes), and one large non-coding region (Control region). The total length of the 13PCGs is 11,216 bp, and these encode 3,727 amino acids, accounting for 73.5% of the complete mitogenome. All the PCGs are initially encoded by ATN and terminated coding with TAA, TAG, or single T. The base composition of the 13 PCGs is 79.4 A + T (A = 33.6%, T = 45.8%, C = 10.1%, and G = 10.5%).

All of the tRNAs were identified by ARWEN version 1.2 software (Laslett and Canbäck 2008), all tRNAs could form a typical cloverleaf structure except *trn-S1*, in which the dihydrouridine arm formed a loop. The lengths of the tRNAs ranged from 60 bp (*trrn-S1*) to 71 bp (*trn-K*). rRNA genes are identified by comparison with *Papilio memnon* (MN013052). The *16S rRNA* gene is 1,322 bp in size and is located between *trn-L1* and *trn-V*; the *12S rRNA* gene is 772 bp in length and is located after *trn-V*. The control region is located between *12S rRNA* and *trn-I* with 457 bp.

**Figure 3.**
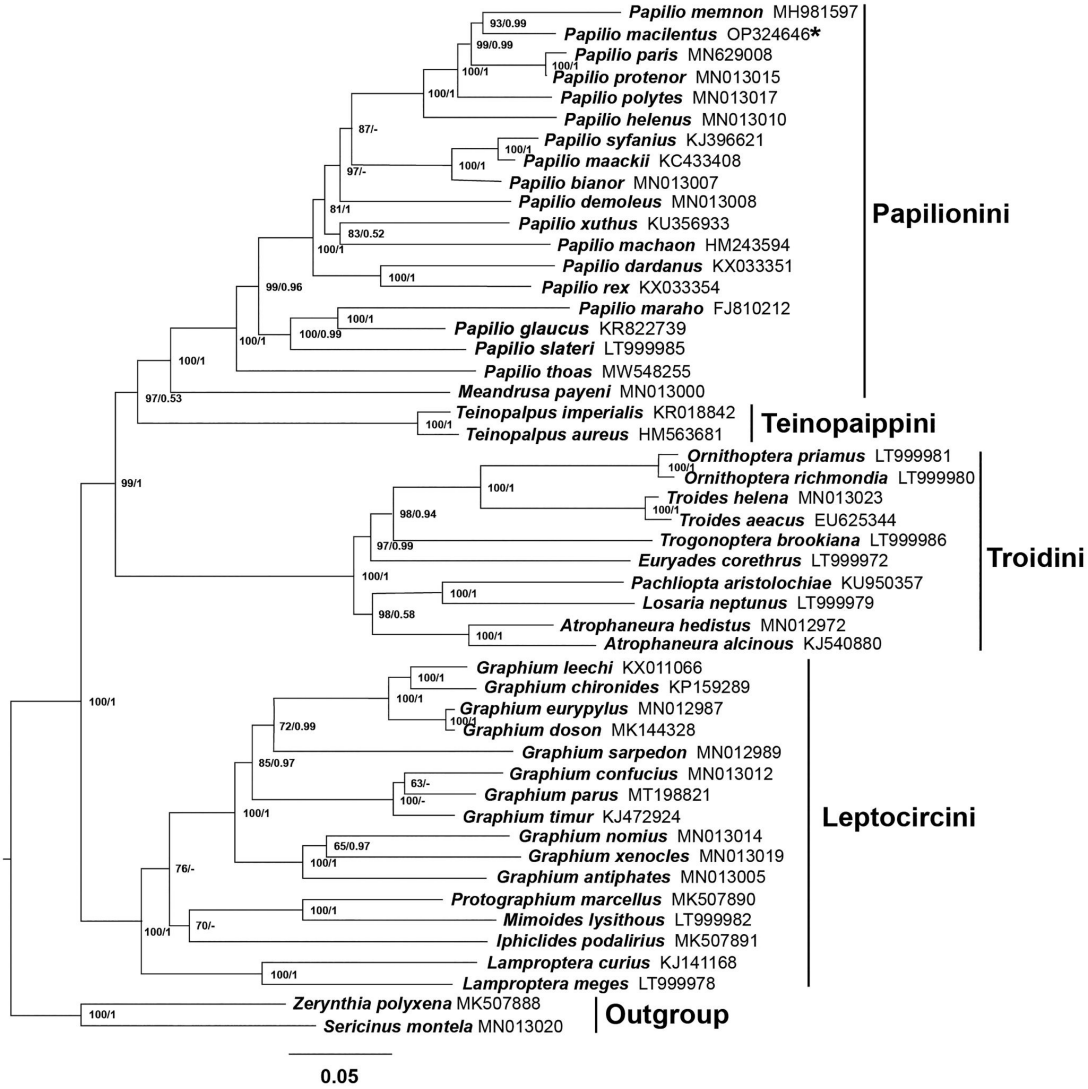
Phylogenetic tree of *Papilio macilentus* and other related species based on 13 PCGs and 2 rRNAs. The numbers at the nodes separated by ‘/’ indicate analyses based on either the ML (left) or BI (right) methods, respectively. ‘-’ are lack of Bayesian posterior probabilities indicates that the nodes were not recovered in the BI analysis. GenBank accession numbers of each mitogenome are given after the species name, and the asterisk indicates the species sequenced in this study. The following sequences were used: *Zerynthia polyxena* MK507888 (Condamine et al. 2018); *Troides helena* MN013023 (Liu et al. 2020); *Troides aeacus* EU625344; *Trogonoptera brookiana* LT999986; *Teinopalpus imperialis* KR018842 (Huang et al. 2015); *Teinopalpus aureus* HM563681 (Qin et al. 2012); *Sericinus montela* MN013020 (Liu et al. 2020); *Protographium marcellus* MK507890 (Condamine et al. 2018); *Papilio xuthus* KU356933 (Wu et al. 2016); *Papilio thoas* MW548255 (Jeng et al. 2021); *Papilio syfanius* KJ396621 (Dong et al. 2016); *Papilio slateri* LT999985; *Papilio rex* KX033354; *Papilio protenor* MN013015 (Liu et al. 2020); *Papilio polytes* MN013017 (Liu et al. 2020); *Papilio paris* MN629008 (Sun et al. 2020); *Papilio memnon* MH981597 (Shi et al. 2020); *Papilio maraho* FJ810212 (Wu et al. 2010); *Papilio machaon* HM243594; *Papilio maackii* KC433408 (Dong et al. 2013); *Papilio helenus* MN013010 (Liu et al. 2020); *Papilio glaucus* KR822739 (Cong et al. 2015); *Papilio demoleus* MN013008 (Liu et al. 2020); *Papilio dardanus* KX033351; *Papilio bianor* MN013007 (Liu et al. 2020); *Pachliopta aristolochiae* KU950357; *Ornithoptera richmondia* LT999980; *Ornithoptera priamus* LT999981; *Mimoides lysithous* LT999982; *Meandrusa payeni* MN013000 (Liu et al. 2020); *Losaria neptunus* LT999979; *Lamproptera meges* LT999978; *Lamproptera curius* KJ141168 (Qin et al. 2015); *Iphiclides podalirius* MK507891 (Condamine et al. 2018); *Graphium xenocles* MN013019 (Liu et al. 2020); *Graphium timur* KJ472924 (Chen et al. 2016a); *Graphium sarpedon* MN012989 (Liu et al. 2020); *Graphium parus* MT198821 (Duan et al. 2020); *Graphium nomius* MN013014 (Liu et al. 2020); *Graphium leechi* KX011066; *Graphium eurypylus* MN012987 (Liu et al. 2020); *Graphium doson* MK144328 (Kong et al. 2019); *Graphium confucius* MN013012 (Liu et al. 2020); *Graphium chironides* KP159289 (Chen and Hao 2016); *Graphium antiphates* MN013005 (Liu et al. 2020); *Euryades corethrus* LT999972; *Atrophaneura hedistus* MN012972 (Liu et al. 2020); *Atrophaneura alcinous* KJ540880 (Chen et al. 2016b).

The resulting tree was represented and edited using FigTree v1.4.1 (Figures S2 and S3). The two topologies are highly similar (Figure 3). Both trees support the monophyly of each tribe of Papiliononae with high bootstrap among almost all nodes. Papilionini and Teinopaippini were sister groups and then clustered with Troidini and eventually converged with Leptocircini. The result also indicated that *P. macilentus* has the closest relationship with *P. memnon* and is clustered within Papilionini clade.

## Discussion and conclusion

In this study, the composition and structure of *P. macilentus* mitogenome are predicted and analyzed, and the features are consistent with those of other *Papilio* insects, including the general genome size, AT content, and gene arrangement (Zuo et al. 2018). ML and BI analysis produced two consensus structures on tribe level, with the topology (((Papilionini + Teinopaippini)+Troidini)+Leptocircini), and this topology was affirmed by Miller’s research (1987) but in contrast to the later hypothesis, which believed that the Papilionini and Troidini formed a sister relationship, and then clustered with Teinopaippini (Simonsen et al. 2011). The monophyly of the tribe Papilionini was recovered in this research, the sister relationship of *Papilio* and *Meandrusa* has also been confirmed in previous studies (Yan et al. 2022). The phylogenetic status of *Papilio demoleus* in Papilionini and *Iphiclides podalirius* in Leptocircini is unclear. Otherwise, the relationships among *Graphium parus*, *G. confucius*, and *G. timur* were inconsistent by ML and BI analysis. These inconsistent analysis results may be due to insufficient sample size and the limited information sites of mitochondrial genes. This research will provide basic data for phylogenetic analysis and population genetic diversity protection of Papilionidae in the future.

## Supplementary Material

Supplemental Material

## Data Availability

The mitochondrion genome sequence data that support the findings of this study are openly available in GenBank of the National Center for Biotechnology Information (NCBI) at https://www.ncbi.nlm.nih.gov/ under the accession no. OP324646. The associated BioProject, Bio-Sample numbers, and SRA are PRJNA1044041, SAMN38366985, and SRR26915771, respectively.
