# Supplementary material for: Complete mitochondrial genome data and phylogenetic analysis of Papilio macilentus Janson, 1877 (Lepidoptera: Papilionoidea: Papilionidae)
Source: Mitochondrial DNA B Resour. 2024 May 13;9(5):631–5. doi: 10.1080/23802359.2024.2351536 (PMC11095290; doi:10.1080/23802359.2024.2351536)
Supplement: Supplemental Material [file TMDN_A_2351536_SM8135.docx]

**Complete mitochondrial genome data and phylogenetic analysis of** ***Papilio macilentus*** **Janson, 1877 (Lepidoptera: Papilionoidea: Papilionidae)**

**Yun-Fei Wu,** **Wei-Hao Yang, Ai Jin, Yan Dong, Jia-Jia Wang*, Li-Xin Zhu***

College of Biology and Food Engineering, Chuzhou University, Chuzhou, China

***Corresponding author:** Jia-Jia Wang (wjchuzhou01@126.com), Li-Xin Zhu [(](mailto:(wjchuzhou@126.com))[czbio01@126.com)](mailto:(wjchuzhou@126.com)).

**Supplementary material**


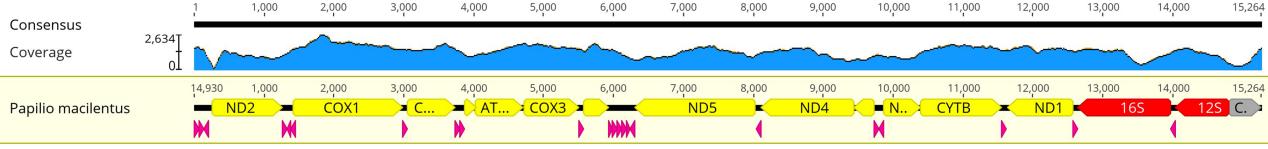


Figure S1. The coverage-depth map of *Papilio macilentus* mitogenome.


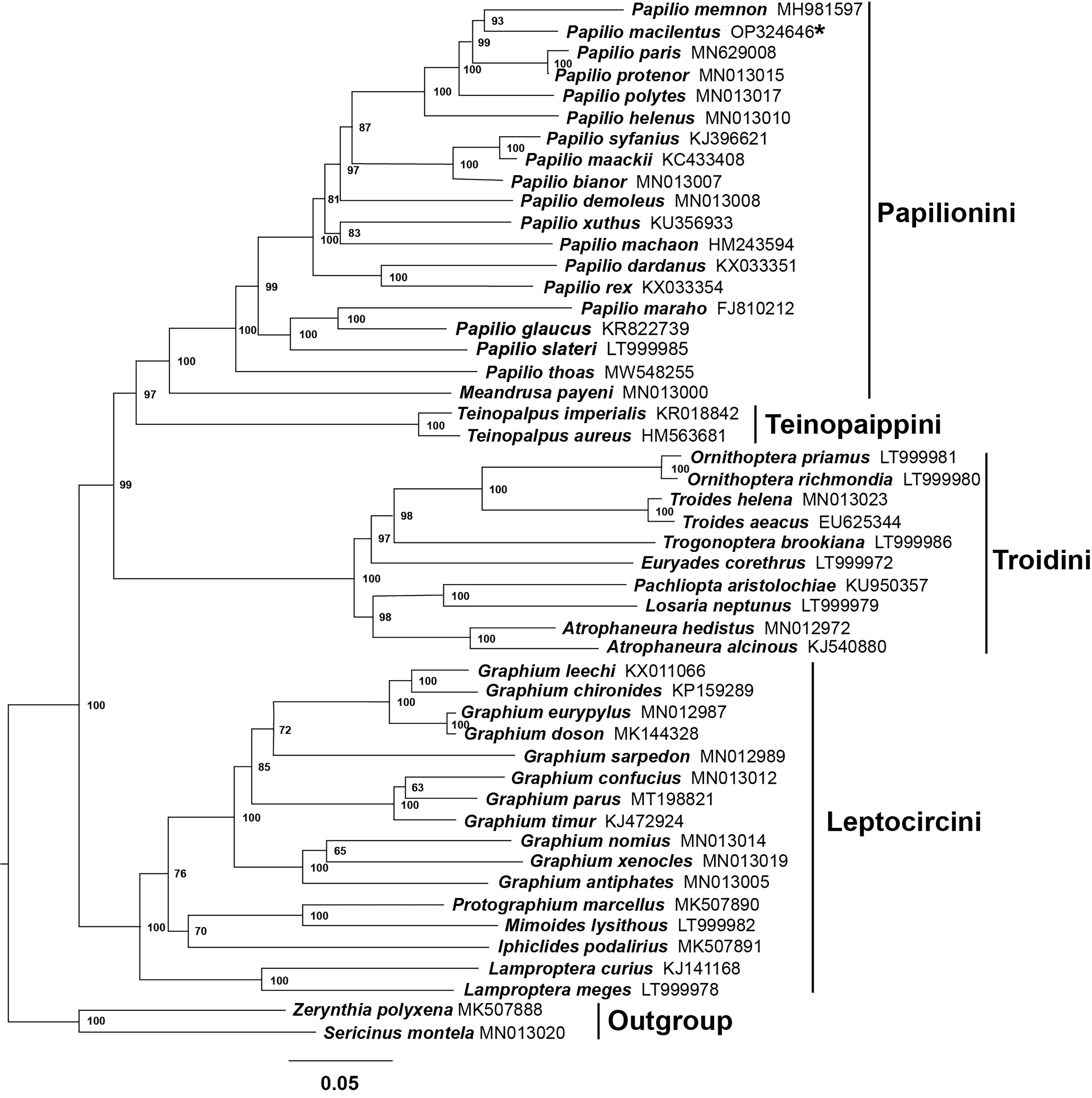


Figure S2. Phylogenetic tree inferred from ML methods based on 13 PCGs and 2 rRNAs.


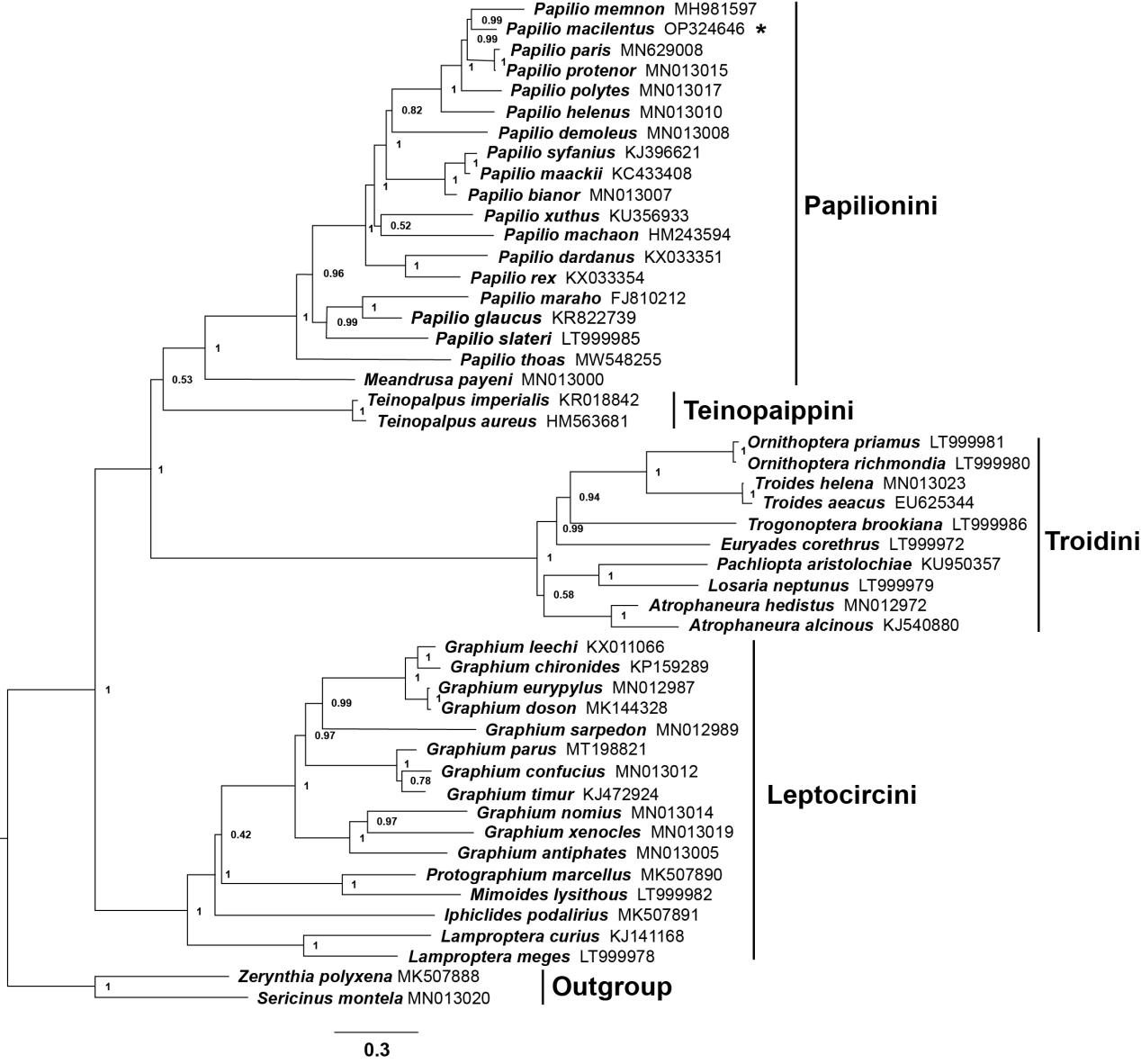


Figure S3. Phylogenetic tree inferred from BI methods based on 13 PCGs and 2 rRNAs.
